# Advanced Imaging for Detection of Foci of Infection in *Staphylococcus aureus* Bacteremia- Can a Scan Save Lives?

**DOI:** 10.1053/j.semnuclmed.2023.01.002

**Published:** 2023-03

**Authors:** Anna L. Goodman, Alice Packham, Amy R. Sharkey, Gary J.R. Cook

**Affiliations:** ⁎ Medical Research Council Clinical Trials Unit at University College London, UK; † Department of Infection, Guy's and St Thomas’ NHS Foundation Trust, London, UK; ‡ Clinical Research Facility, University Hospitals Birmingham Foundation Trust, London, UK; § Department of Cancer Imaging, School of Biomedical Engineering and Imaging Sciences, King's College London, London, UK; ║ Department of Radiology, Guy's and St Thomas' NHS Foundation Trust, London, UK; ¶ King's College London and Guy's and St Thomas’ PET Centre, Guy's and St Thomas' NHS Foundation Trust, London, UK

## Abstract

Bloodstream infection or sepsis is a common cause of mortality globally. Staphylococcus aureus (S. aureus) is of particular concern, through its ability to seed metastatic infections in almost any organ after entering the bloodstream (S. aureus bacteraemia), often without localising signs. A positive blood culture for S. aureus bacteria should lead to immediate and urgent identification of the cause. Failure to detect a precise focus of infection is associated with higher mortality, sometimes despite appropriate antibiotics.

This is likely due to the limited ability to effectively target therapy in occult lesions. Early detection of foci of metastatic S. aureus infection is therefore key for optimal diagnosis and subsequent therapeutic management. ^18^F-FDG-PET/CT and MRI offer us invaluable tools in the localisation of foci of S. aureus infection. Crucially, they may identify unexpected foci at previously unsuspected locations in the body, for example vertebral osteomyelitis in the absence of back pain. S. aureus bloodstream infections are further complicated by their microbiological recurrence; ^18^F-FDG-PET/CT provide a means of localising, thus enabling source control.

More evidence is emerging as to the utility of ^18^F-FDG-PET/CT in this setting, perhaps even to the point of reducing mortality. ^18^ F-FDG-PET/MRI may have a similar impact. The available evidence demonstrates a need to investigate the impact of ^18^F-FDG-PET/CT and MRI scanning in clinical management and outcomes of S. aureus infection further in a randomised prospective clinical trial.

## Introduction

Bloodstream infection or septicemia is a life-threatening condition which accounts for up to one in five deaths world-wide.^1^ Even with appropriate treatment, once bacteria are present in the bloodstream they can locate at a particular site or organ which can then be more challenging to treat effectively.^2^ Should these sites remain occult this negatively affects patient outcomes, probably due to the inevitable impact on our ability to then target therapy effectively.^3^ *Staphylococcus aureus* bacteremia (SAB) may commonly present with an occult site of focal infection and can seed infectious foci widely: for example, the heart (endocarditis), bone, and joints (osteomyelitis and septic arthritis), lung or pleura (pneumonia, empyema), surgical wounds, skin, and soft tissue infections (cellulitis, myositis). Infection can occur at multiple sites in the same individual. Foci can be associated with a removable device, for example, peripheral or central venous catheters; or devices where removal is more challenging, such as a prosthetic joint or heart valve.^3^ Advanced imaging methods such as ^18^F-FDG-PET/CT and MRI can be used to detect foci that may not be suspected clinically.^4^ In turn, this may enable us to better target therapy: to drain an abscess, remove an infected prosthesis, or correctly prescribe antibiotics to penetrate a particular organ, through which we may improve clinical outcomes.

## How Does *Staphylococcus aureus* Lead to Foci of Infection?

In up to 20% of *S. aureus* infections, the site of infection is classed as unknown. Such a classification is associated with higher mortality, suggesting the importance of establishing infection location through considered investigation and imaging. The ability of advanced imaging to scan the whole body in one sitting is a clear advantage when targeting *S. aureus* infection, considering the wide range of body systems *S. aureus* may infect.

### Local Spread From Colonization

**Figure 1:**
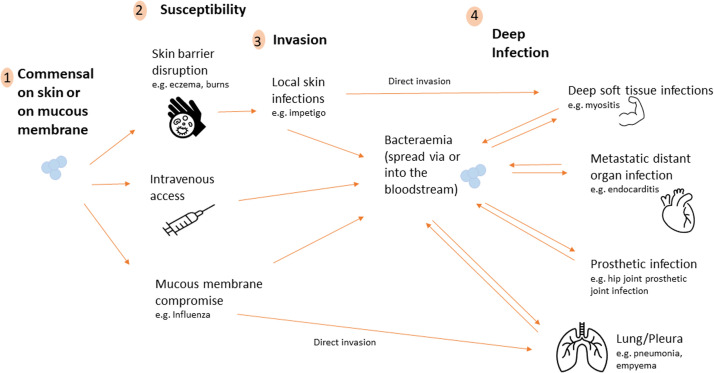
Mechanism of *Staphylococcus aureus* spread leading to deep and disseminated infection. (Color version of figure is available online.)

To understand the potential utility of ^18^F-FDG-PET/CT in *S. aureus* bloodstream infection it is important to appreciate how these bacteria spread, leading to deep infection (Fig. 1). *S. aureus* lives harmlessly on (colonizes) our skin and in the nasal passages constantly in approximately 20% of adults and intermittently in a further 30% of adults.^5^ Disruption may lead to local infection in the skin or nose, and to nearby infections such as furunculosis or sycosis barbe (hair follicles), impetigo (skin), and blepharitis (eyelids). *S. aureus* may then spread directly from such localized infections to deeper body sites. When natural protective barriers are compromised, such as during device insertion or skin breakdown, the spread may occur via these means into the bloodstream. Spreading through mucosal membranes may occur when they are compromised, such as during influenza, leading to pulmonary invasion and severe pneumonia. Pleural effusions associated with pneumonia can become secondarily infected leading to empyema.

**Figure 2:**
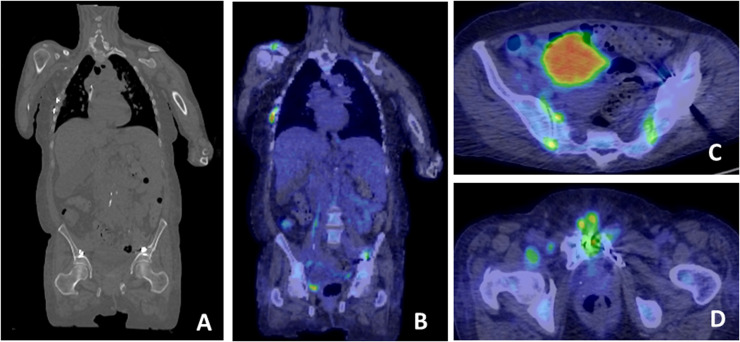
^18^F-FDG-PET/CT used to reveal occult foci of infection ^18^F-FDG-PET/CT was used to look for occult foci of infection in a case of recurrent *S. aureus bacteraemia* (A) This shows permanent implanted metal work in the right ribs and in the pelvis (B) This reveals possible foci of FDG avid infection at the right acromioclavicular joint and right ribs (C) FDG-avid bilateral sacroiliitis D: FDG-avid osteomyelitis of the pubic symphysis associated with metalwork. (Color version of figure is available online.)

### Disrupted Skin Barriers

Skin pathologies, such as eczema, burns, or skin disruption from ulceration, increase the chance of blood contamination during sampling. This can rarely lead to false positive blood cultures, suggesting SAB when bloodstream infection is not present. However, patients with skin pathologies are at higher risk of bloodstream infection as their skin barrier provides less defense to infection. Thus it can be difficult to differentiate SAB and contamination in a patient with a fever and disrupted skin.

### Direct Tissue Invasion

Tissue invasion can be particularly severe with virulent strains of *S. aureus* such as Panton Valentine Leucocidin (PVL) and/or methicillin-resistant S. aureus (MRSA). When these types of *S. aureus* enter the bloodstream the clinical outcomes are increasingly poor.^6^ As such, foci detection and directed treatment must be prioritized. PVL acts to suppress the local immune response, resulting in deep skin infection and large abscesses, requiring incision and drainage. PVL also characteristically causes necrotic lung abscesses and cavities. Such cavitations are visible on chest radiography or CT, appearances that should lead to consideration of *SAB* in patients without a prior diagnosis. MRSA may also form abscesses, with the additional challenge of drug resistance.^7^

### Haematogenous Spread (Blood)

*S. aureus* can spread to numerous body sites via the blood, including lung, bone, muscle, heart valves, and brain. The infection pattern in those who inject drugs, in whom SAB is common, may be lung abscesses and right-sided endocarditis due to the anatomy of bacterial spread through the venous system. In situ deep musculoskeletal infection with *S. aureus* can occur; so-called "tropical" myositis, pyomyositis, or purulent infectious myositis was initially reported in greater prevalence in tropical climates, including East African countries such as Uganda. There have been increasing reports of *S. aureus*-associated myositis in the United States in the last few decades, linked to immunocompromised states such as human immunodeficiency virus, diabetes mellitus and hematological malignancies.^8^ Muscle, skin, and soft tissue infections would be expected to be more common in diabetes, due to the tissue hyperglycemia providing an ideal growth environment for bacteria.

### Prosthetic Devices

If the skin and mucosal lining integrity are established the next question in the radiological assessment of a patient with bloodstream *S. aureus* is whether prosthetic material of any kind is present. This may include a temporary peripheral venous catheter, on which colonized skin *S. aureus* may pass through the skin barrier during the insertion of prosthetic material. The bacteria can reside on artificial material through the generation of biofilms. Alternatively, once artificial material is inserted into the body, an infection at another site, such as a skin abscess, may temporarily enable *S. aureus* to travel hematogenously to infect a previously uninfected prosthetic area. This enables the infection to spread to implanted metalwork such as in joints and heart valves, leading to a prosthetic joint infection or prosthetic valve endocarditis (PVE) respectively.

Some devices simply cannot be removed, or the procedures required to remove them would be such a high risk in a frail patient to consider that approach impossible. However, if left untreated the organism is aggressive and subsequent spread and problems are likely. Failure to remove an infected intravascular device was the most important risk for treatment failure in one study which found, after controlling for other factors, patients whose intravascular device was not removed were 6.5 times more likely to relapse or die of their infection than those whose device was removed.^9^ Such problems can recur for many years, with patients presenting on multiple occasions with recurrent life-threatening episodes of *S. aureus* bloodstream infection secondary to the in-situ device. These organisms are more likely to be a hospital-acquired type, which is often more complex to treat, with higher rates of antibiotic resistance; hospital-acquired MRSA tend to be resistant to more antibiotics than community-acquired MRSA. ^18^F-FDG-PET/CT can be used to determine whether a device is infected, and its utility to identify prosthetic foci is demonstrable through new guidelines recommending its use. ^18^F-FDG-PET/CT is routinely recommended in vascular infection guidelines, for example,^10^ aiding surgical management decisions as to the practicalities and necessity of device removal.

In summary, *S. aureus* leads to foci of infection through a combination of colonization, direct spread through disrupted natural anatomical barriers, hamatogenous spread, and potentially surgical biofilms. The mechanism of spread is likely to determine the foci of infection.

## Can ^18^F-FDG-PET/CT be Used to Detect Foci of Infection in *S. aureus* Bloodstream Infection (Bacteremia, SAB)?

^18^F-FDG-PET/CT is increasingly used in the detection of infection, due to its ability to detect inflammation and infection, in the form of increased glycolytic metabolism in activated neutrophils, lymphocytes, and macrophages.^11^ The degree of utility of ^18^F-FDG-PET/CT imaging in SAB depends on the true focus of infection. If the focus is single and known before imaging (such as a prosthetic heart valve in endocarditis) then the test may be unnecessary. However, alternatively, it may also expose previously unidentified second sites of infection, which require separate, distinct management. In those with a prosthetic aortic graft, there are clear guidelines to use ^18^F-FDG-PET/CT, suggesting a clear role if there is a concern of infection.^6^ New guidelines for infective endocarditis incorporate the need for ^18^F-FDG-PET/CT in this condition.^12^

A recent single center matched cohort-study from Israel suggests ^18^F-FDG-PET/CT should be performed on all patients with SAB.^13^ They compared the outcomes of 149 prospectively recruited patients with *SAB* who underwent ^18^F-FDG-PET/CT to a control group of 150 matched patients with SAB who had not undergone ^18^F-FDG-PET/CT. Using a primary outcome of 90-day mortality they found that the mortality in those receiving ^18^F-FDG-PET/CT was significantly lower than in their matched control group (13.9% vs 28.5%, *P* = 0.002), concluding ^18^F-FDG-PET/CT was independently associated with lower mortality. Patients in the intervention group had longer treatment durations and underwent a higher number of focus control procedures, such as drainage of collections found on ^18^F-FDG-PET/CT, providing a possible mechanistic explanation of the finding of improved survival. An element of referral bias cannot be excluded due to the use of a retrospective control cohort, and the higher rate of echocardiography occurring alongside ^18^F-FDG-PET/CT in the prospective group may have been a confounder. As ^18^F-FDG-PET/CT is a diagnostic tool rather than a direct intervention, its impact will inevitably have a multifactorial basis which cannot be analyzed as a direct causation. However, these promising findings indicate a positive impact of ^18^F-FDG-PET/CT when used to detect foci of infection in SAB.

TEPSTAR is a current RCT (NCT03419221) in France prospectively exploring ^18^F-FDG-PET/CT vs usual care in 290 adults with SAB (approx. 200 patients currently recruited, expected recruitment completion January 2024). The primary outcome measure is based on the detection of foci of infection and this trial seeks to assess the management impact of ^18^F-FDG-PET/CT in this setting. We await the results with interest.

## Is There a Particular Subgroup of Those With *SAB* Who Particularly Benefit From ^18^F-FDG-PET/CT? Complicated or Uncomplicated, High- or Low-Risk?

^18^F-FDG-PET/CT is becoming established in having a role in changing management in SAB. The likelihood of a management change, such as a change in antibiotic duration, following ^18^F-FDG-PET/CT appears to be of the order of 16%-19%.^13^, ^14^, ^15^, ^16^ This compares with the degree of adjustment of dose or change in the duration of therapy of 16%-19% seen in malignancy.^17^ A study in 1998 sought to differentiate those patients with SAB at high and low risk of an adverse outcome and stratify their treatment accordingly.^9^ They referred to ‘simple,’’ ‘‘uncomplicated,’’ ‘‘endocarditis,’’ and ‘‘extracardiac.’’ SAB and proposed different durations of intravenous antibiotic treatment for the different conditions (7 days, 14 days, 4-8 weeks, and 4-8 weeks, respectively). A ‘‘simple’’ SAB had rapid clinical resolution (within 72 hours of initiating therapy and removal of focus), a negative transoesophageal echo (TOE) for both vegetations and predisposing valve abnormalities, negative repeat blood cultures (showing clearance of bacteria from the blood) at day 2-4 after starting appropriate treatment, and a removable focus of infection with no remaining indwelling prosthetic devices. ‘‘Uncomplicated’’ SAB described those with predisposing valvular abnormalities but no vegetation on TOE, a positive blood culture despite treatment, a superficial nonremovable focus of infection or ongoing signs of infection after 72 hours of appropriate antibiotics. Given the high mortality of this condition, treatment courses have since been extended in the ‘‘uncomplicated’’ group, which now would be considered ‘‘complicated’’ due to the presence of bloodstream bacteria following 2 days of appropriate treatment. In MRSA SAB, the Infectious Diseases Society of America no longer uses the term simple, and define uncomplicated through the absence of positive follow-up blood cultures. In fact, their description of uncomplicated MRSA SAB appears similar to that of simple SAB but with the addition of the term ‘‘no metastatic infection. ’’^18^ ^18^F-FDG-PET/CT is an obvious tool for clinicians to utilize in the exclusion of metastatic infection in determining the duration of treatment in such cases.

## Gram Positive Organisms and Metastatic Infections- ‘‘High-Risk Patients’’

A Gram-positive organism has a thick peptidogycan cell wall, which stains purple by Gram stain for microscopy investigations. These may be visible in clusters (usually Staphylococci) or chains (usually Streptococci). An early study in 2010 sought to explore the role of ^18^F-FDG-PET/CT in a range of Gram-positive infections in the Netherlands.^19^ Notably, other Gram-positive organisms, such as *Enterococcus sp.* and *Streptococcus*, have similar infectious patterns to *S. aureus*, including spread to, and through, the bloodstream. Some Gram-positive organisms were excluded, such as coagulase-negative *Staphylococci* (eg, *Staphylococcus epidermidis*- this group does not include *S. aureus* which is coagulase-positive), which behave differently. This prospective study found the majority of those recruited (64% of cases and 64% of controls) had *S. aureus* bacteremia. They included 115 patients who had a PET/CT scan and only included those patients considered to be at higher-risk of metastatic infection due to their stratification (Table). The control group was retrospective (those with similar features admitted 2000-2004) whilst those having a ^18^F-FDG-PET/CT were prospective and admitted 2005-2008. This makes it hard to interpret; it is possible that changes in clinical management between these years impacted patient outcomes in each cohort, such as increased infection specialist consultations at the bedside or changes in antimicrobial recommendations. They concluded that performing a ^18^F-FDG-PET/CT within 14 days of the first positive blood culture in a patient with ‘‘high-risk’’ gram positive bacteremia was associated with significantly lower 3-month mortality (19% with PET/CT vs 32% without PET/CT, *p* = 0.01). This study also found that antibiotic therapy was changed as the result of the ^18^F-FDG-PET/CT result in 15% of cases and that alternate new diagnoses were established in 10%.

**Table:** High-risk criteria used^15^,^19^

Community acquisition of infection
Signs of infection for >48 hours before appropriate treatment was initiated
Fever for >72 hours after the initiation of appropriate treatment
Positive blood cultures more than 48 hours after the initiation of appropriate treatment

## SAB in High-Risk Patients

A subsequent single-center retrospective observational study sought to investigate this question further and specifically focused on *S. aureus.*^15^ They reviewed 196 cases of *S. aureus* bloodstream infection but again considered that ^18^F-FDG-PET/CT was only presumed necessary in those at ‘‘high-risk’’- of whom they found 102 cases. Approximately half of that group had received a ^18^F-FDG-PET/CT in their routine care and they found a significantly lower mortality at 1 year in those who did (17% vs 44%, *p* = 0.002). However, it is possible the higher mortality represents the palliative path of those who did not receive a ^18^F-FDG-PET/CT as they were deemed too unwell for such an investigation.

In summary, the most recent trial provides the strongest evidence of benefit from ^18^F-FDG-PET/CT. This trial suggests that both ‘‘high’’ and ‘‘low’’ risk patients with *S. aureus* bloodstream infection should be routinely offered ^18^F-FDG-PET/CT, demonstrated through the mortality benefit in the ^18^F-FDG-PET/CT cohort, which remained significant in subgroups with community-onset infections without malignancy and amongst patients who were not considered to be ‘‘high-risk’ before their scan (Table).^13^ However, this requires further investigation and evidence, particularly to justify ionizing radiation and cost for patients whose conditions may be considered a lower risk of poor outcomes.

## Are There Additional Factors That Need to be Considered With ^18^F-FDG- PET/CT in *S. aureus* Bacteremia?

### Patient Transfers

When considering the utility of ^18^F-FDG-PET/CT in SAB, the clinician must first determine the condition of the patient. Septicemia is an acute illness and patients may be clinically unstable, with hemodynamic instability or an intensive care ventilation requirement. Transfer to a PET scanner, which may be off-site or in another hospital, may be too challenging or risky for that patient at the time it is most needed. Alternatively, the decision may be made to do the ^18^F-FDG-PET/CT once a patient is clinically stabilized.

### Timing

The timing of ^18^F-FDG-PET/CT in SAB is complex. ^18^F-FDG-PET/CT is likely to have higher sensitivity for foci earlier in the infection as antibiotics may reduce the uptake of ^18^F-FDG in infectious foci. In addition, an earlier scan will provide information earlier in a hospital stay, thus providing more time to act to control a source. However, a known source of infection may disappear before a ^18^F-FDG-PET/CT and this might be a benefit as it demonstrates effective treatment. In general, the first 7-14 days is felt to be the ideal timing of a ^18^F-FDG-PET/CT to detect foci in time to intervene but the scan may be helpful later particularly when there is an outstanding clinical concern or failure to respond. Ideally, a TOE would also be performed in these first 7-14 days though as an interventional procedure, this is not always possible or may be rejected by the patient. The benefit of early intervention is to optimize the antibiotic management with personalized therapeutics through decision-making support for antibiotic duration or source drainage or removal procedures.

### Diet and Diabetes

The protocol for ^18^F-FDG-PET/CT in *S. aureus* bacteremia requires a fast or modified diet.^20^ A minimum fast of 4-6 hours is required but if a cardiac source is possible then a low-carbohydrate, fat-allowed diet in the 24 hours preceding ^18^F-FDG-PET/CT scan is recommended to minimize physiological myocardial activity, with any glucose- or insulin-containing infusions discontinued in the 4-6 hours preceding the scan. Radiolabelled ^18^F-FDG, the standard PET/CT tracer, is a glucose analog. Therefore, strict glucose control is preferred to ensure optimal contrast between pathology and normal organ activity. The requirement for the glucose levels to be 4-10 mmol/L to obtain an optimal ^18^F-FDG-PET/CT is complex in the setting of SAB. Logistically, this requires intensive blood glucose ward-based monitoring and poses communication and implementation challenges of facilitating fasts and modified diets in a hospital setting. When considering patient risk factors, those living with diabetes have a higher incidence of *S. aureus* bacteremia. Glucose is particularly difficult to regulate in this cohort during SAB infection; which causes increased tissue hyperglycemia combined with decreased oxygenation and reduced immunity. In addition, capillary blood glucose levels are likely to rise during acute illness. However, it is likely that a focus on optimal glucose control in preparation for a ^18^F-FDG-PET/CT would have other benefits in the unwell patient and is worth the additional patient input and complications of fasting.

### Diet and Endocarditis

Whilst a 4-6- hours fast is standard when cardiac lesions are not suspected, a more prolonged fasting protocol may be required to adequately suppress myocardial activity in patients with a suspected cardiac focus as ^18^F-FDG-PET/CT may otherwise not routinely detect cardiac involvement. To optimally assess native cardiac valves 12 hours or more of fast are required in addition to a more prolonged low carbohydrate diet in order to minimize the background myocardial uptake of tracer.^21^ Heparin has also been found to further suppress the myocardial tracer uptake.^22^ This can make an already complex procedure even more challenging but the incidence of over 20% of endocarditis in these cases means that the logistic challenges may be worthwhile. The possibility of false negative results is high, particularly in native valve endocarditis and this should always be combined with transthoracic echocardiography (TTE) and ideally TOE. False positive ^18^F-FDG-PET/CT may also suggest native valve endocarditis (NVE) where it is not present, when increased ^18^F-FDG uptake can be detected inside and outside the heart from other causes of inflammation, or when the myocardium is inadequately suppressed, particularly in earlier studies where fasting was not prolonged.^23^ False positive ^18^F-FDG-PET/CT scans are particularly an issue when looking for PVE as inflammation associated with the surgical field and even the adhesive used at operation may potentially cause tracer uptake.^24^ There is growing evidence of the role of ^18^F-FDG-PET/CT in detecting endocarditis and cardiac infection.^25^ In a systematic review of 509 cases of endocarditis the sensitivity and specificity of ^18^F-FDG-PET/CT in endocarditis were improved as fasts were prolonged and the scanners improved in speed, image quality, and accuracy after 2015, and were 74% and 88% overall.^23^ The authors found higher sensitivity for PVE (86%) and cardiac implantable electronic devices (CIED infection) (72%) than NVE (31%) while specificity was over 70% in all cases. This had improved from an earlier systematic review which found ^18^F-FDG-PET/CT detected just 14% of NVE with an appropriate pre-scan diet and even just 6% without^26^ for PVE and CIED infection ^18^F-FDG-PET/CT can be expected to be both sensitive and specific for the source of *S. aureus* bacteremia. In contrast in NVE ^18^F-FDG-PET/CT is unlikely to detect all cases, but when it does this would be expected to be a valvular infection. Due to its greater efficacy for PVE, ^18^F-FDG-PET/CT has recently been incorporated into the European guidelines for diagnosis of PVE and CIED infection^27^ whilst American guidelines now suggest it be used in a wider range of endocarditis, including NVE.^12^

## Vascular Access and ^18^F-FDG-PET/CT

Other risk factors in *S. aureus* bacteremia are a disruption to blood vessels. This may occur through the injecting of recreational drugs and the use of vascular access devices, as commonly occurs in end-stage renal failure. In both these scenarios there may be very difficult vascular access. This can impact the practicalities of ^18^F-FDG-PET/CT scanning.

### Scan Anatomy Considerations

^18^F-FDG-PET/CT scans are usually performed from the base of the skull to the upper thighs. A key differential for *S. aureus* bacteremia in a patient with diabetes would be a diabetic foot infection. It is critical that the feet are inspected prior to the submission of a scan request and there is consideration of this possibility- one option being to routinely include imaging of the feet in those known to have diabetes or who have vascular disease, another key risk factor for a diabetic foot infection. Those with diabetes are also at higher risk of skin and soft tissue infection and this may alternatively be detected in a previously unsuspected area, having perhaps been missed on a clinical examination if full exposure has not occurred.

## ^18^F-FDG-PET/CT in the Recurrence of *S. aureus* Bacteremia

Recurrence of *S. aureus* bacteremia can occur days, weeks or even years after an initial episode. The challenges in detecting infectious foci clinically can lead to caution with prolonged treatment used to prevent relapse. ^18^F-FDG-PET/CT can be used to reassure the clinician that there is no infection remaining during or following treatment in uncomplicated cases. Alternatively, for example in the setting of a prosthesis infection in which the device cannot be removed for clinical or anatomical reasons, it can provide information on the likelihood of ongoing infection to be utilized in calculating operative risk vs the risk of the device remaining. The importance of device removal needs to be highlighted in this setting as ongoing bacteremia can lead to seeding to secondary sites- for example, a persistent prosthetic joint infection can then subsequently lead to an infection of a remote native joint. In this setting, the likely location of infection may be known. Graft infections are routinely assessed with ^18^F-FDG-PET/CT in order to differentiate between graft changes and infective changes radiologically.^10^ Large vascular grafts may be very challenging to remove and thus there may be a situation of monitoring an area over time via ^18^F-FDG- PET/CT to determine if there is ongoing infection or recurrence.

## ^18^F-FDG-PET/CT in Pediatric Populations

*S. aureus* infection is one of the most frequent diagnoses identified when a pediatric infectious disease physician is consulted.^28^ Specific risk factors for pediatric SAB include young age (particularly neonates), comorbidities, socioeconomic factors, and the presence of a central venous catheter.^6^^,^^29^ Thirty-day mortality rates from pediatric SAB, while lower than in adult populations, remain significant, reported as between 4.7% and 21%.^30^^,^^31^

Evidence-based definitions for severe pediatric SAB have not been defined due to the current lack of high-powered trial data with quantifiable outcome measures. Higher rates of intensive care admission and mortality have been reported from observational study data in septic patients without a diagnosed infectious focus. Therefore, as with adult SAB patients, accurate, early diagnosis of SAB foci in pediatric patients is vital to target treatment and improve outcomes.

SAB behaves differently in pediatric populations. Diagnosis must consider location affinities of SAB in pediatrics, alongside challenges of imaging in children. Skeletal foci, such as osteoarticular infections, have been reported at higher rates in children, at approximately 30% of presentations^31^ There is high-risk that pediatric osteomyelitis develops into chronic or recurrent infections if not diagnosed early. Contrarily, reports of endocarditis are scarce in children without structural cardiac disease (although endocarditis has been identified as a significant focus location in SAB in pediatric patients with underlying congenital heart disease, at 29.5% of presentations).^32^

As with adults, pediatric imaging in SAB may provide significant insight into foci locations, and therefore better direct treatment. However, there are additional considerations of imaging in pediatric populations, including risk vs benefit of radiation exposure, due to the higher pediatric sensitivity to ionizing radiation, and logistical challenges of imaging children. MRI is a widely acceptable imaging option in pediatrics, with the benefit of no ionizing radiation exposure and high anatomical detail. Limitations include the relatively long scan duration, during which the patient must remain still, which often requires sedation or general anesthesia in the pediatric population. PET/CT is only marginally quicker, taking around 20 minutes, and may still require anesthetic input depending on the child. However, there is growing interest in whether ^18^F-FDG-PET/CT may improve diagnostic rates in pediatric bacteremia.

To the best of our knowledge, there are limited large-scale trial data of the use of ^18^F-FDG-PET/CT in pediatric populations with confirmed bacteremia. Retrospective studies in pediatric patients with fever of unknown origin, and suspected infection or inflammation, have identified promising diagnostic results when ^18^F-FDG-PET/CT has been utilized. Pijl et al.^33^ reported that 48% (53 of 110) of ^18^F-FDG-PET/CT scans identified a cause for pediatric fever of unknown origin, with 53% (58 of 110) undergoing a treatment modification following ^18^F-FDG-PET/CT. Endocarditis, juvenile idiopathic arthritis and inflammatory bowel disease were listed as the most common causes but they did not report the microbiology so it is unclear if the endocarditis was SAB or another organism. For use of ^18^F-FDG-PET/CT, they reported a sensitivity of 85.5%, specificity of 79.2%, PPV of 84.1%, and NPV of 80.9%. Ropers et al.^34^ reported on 64 scans from 59 pediatric patients with suspected infection or inflammation. Both reported higher CRP levels in positive scans. Twenty-seven of 64 scans had a positive diagnosis, of which PET/CT contributed to establishing the diagnosis in 85% (23 of 27). It had lower utility in the negative diagnostic scans and contributed towards the final diagnosis by differential narrowing in 38% (8 of 21 scans). Ten of 27 scans in this study revealed an unexpected infection diagnosis and at least 3 of those 10 had *S. aureus* bloodstream infection.

There are potential applications for its select use for high-prevalence foci sites of SAB, such as skeletal infections in pediatric patients. Recent juvenile animal models have reported 100% (20 of 20) detection rates of small osteomyelitis *S. aureus* foci in peripheral bone through PET/CT and outlined comparable identification rates on using the lower injected activity of ^18^F-FDG (falling from 132 MBq down to 4.4MBq).^35^ This has promising applications for maintaining diagnostic accuracy while reducing radiation exposure.

## ^18^F-FDG-PET/MRI

^18^F-FDG-PET can also be used in combination with MRI, allowing ^18^F-FDG-PET /MRI to be performed as a single hybrid scan. ^18^F-FDG-PET/MRI is an emerging technology in cancer diagnosis, combining sensitive cancer detection with high-contrast resolution and detail, shown to allow more accurate staging of tumors.^36^ These advantages of ^18^F-FDG-PET/ MRI could be of use in the detection of occult foci of SAB, particularly if the regular field of imaging (skull vertex to the upper thighs) was extended to the feet, so as not to miss key foci of infection in the lower femur, knees, and feet, particularly in patients with diabetes mellitus. This extended field ^18^F-FDG-PET/MRI would not only enable whole body identification of unsuspected foci of SAB, but would offer a single diagnostic scan, completed in one imaging department visit, compared to the multiple scans required in more circuitous methods to identify SAB complications,^37^ is also associated with reduced ionizing radiation exposure compared to ^18^F-FDG-PET/CT, which is important in younger patients where multiple scans may be required to ensure resolution of infection with appropriate treatment.

The use of single-site ^18^F-FDG-PET/MRI in localized bone and joint infection has been explored when there is a clear focus suspected, and has multiple advantages compared to ^18^F-FDG-PET/CT, including high-resolution soft tissue information. Henkelmann et al.^38^ performed ^18^F-FDG-PET/MRI for the detection of periprosthetic joint infections in 13 prostheses, finding the scan correctly identified every infection (sensitivity 100%, specificity 100%) when compared to the reference standard of surgical or biopsy specimens. This ability was limited to soft tissue identification, and was hampered by difficulty identifying infection within periprosthetic bone due to artefact from the prostheses. A retrospective study by Hulsen et al.^39^ performed single-site ^18^F-FDG-PET/MRI imaging in 36 patients with chronic osteomyelitis, and calculated a sensitivity of 78%, specificity of 100%, and accuracy of 86% sensitivity of ^18^F-FDG-PET/MRI, compared to the ‘‘ground truth’’ (clinical assessment by the orthopedic surgeon that is, based on subsequent intraoperative microbiology or long-term follow-up). ^18^F-FDG-PET/MRI could also overcome some of the challenges of native valve endocarditis diagnosis- we note the small registered study in Edinburgh assessing hybrid ^18^F-FDG-PET/MRI vs ^18^F-FDG-PET/CT in suspected native and prosthetic valve endocarditis and NVE and await the results with interest (NCT03626571).

The limitations of ^18^F-FDG-PET/MRI revolve around the usual limitations of MRI, some of which are pertinent in the detection of SAB: the scan takes approximately one hour to perform and the coils required for MRI can be uncomfortable for those with bone and joint infection, and there is limited resolution around metal prostheses due to artefact, particularly limiting in the setting of suspected prosthetic valve endocarditis or orthopedic prosthetic infection. The single greatest limitation of the fact that very few centers have access to hybrid PET/MRI scanners. To overcome this, MRI could be performed immediately following a ^18^F-FDG-PET/CT (as a ^18^F-FDG-PET/CT plus single-site MRI). However, studies that tried to combine the investigations in SAB found challenges due to the timings required with 25% of patients receiving both MRI and PET/CT on the same day, 56% within 24 hours, and 19% in 24-48 hours.^40^

Whilst ^18^F-FDG-PET/MRI has definite advantages compared to ^18^F-FDG-PET/CT in terms of increased anatomical resolution, high contrast detail and reduced radiation exposure, to determine the diagnostic accuracy in the detection of occult foci of SAB, a dedicated clinical trial would be required to compare the performance of ^18^F-FDG-PET/MRI to ^18^F-FDG-PET/MRI; similar studies have already been performed in oncology trials with the advantage that the both imaging modalities can be undertaken sequentially using a single injection of ^18^F-FDG tracer, with randomization of the order of PET/MR vs PET/CT to avoid bias created by ongoing tracer uptake between the studies.

## Summary/Conclusion

Further prospective randomized studies in this arena would provide an improved understanding of the management impact of these tests in SAB and their cost-effectiveness and clinical utility.
